# Genome-wide analysis of the Thaumatin-like gene family in Qingke (*Hordeum vulgare* L. var. *nudum*) uncovers candidates involved in plant defense against biotic and abiotic stresses

**DOI:** 10.3389/fpls.2022.912296

**Published:** 2022-08-17

**Authors:** Le Wang, Zepeng Xu, Wei Yin, Kai Xu, Shuai Wang, Qianhan Shang, Wei Sa, Jian Liang, Li Wang

**Affiliations:** ^1^State Key Laboratory of Plateau Ecology and Agriculture, Qinghai University, Xining, China; ^2^College of Eco-Environmental Engineering, Qinghai University, Xining, China; ^3^Qinghai Academy of Agricultural Forestry Sciences, Qinghai University, Xining, China

**Keywords:** Tibetan hulless barley, plant immunity, Thaumatin-like protein, expression profile, stress response

## Abstract

Thaumatin-like proteins (TLPs) participate in the defense responses of plants as well as their growth and development processes, including seed germination. Yet the functioning of *TLP* family genes, in addition to key details of their encoded protein products, has not been thoroughly investigated for Qingke (*Hordeum vulgare* L. var. *nudum*). Here, a total of 36 *TLP* genes were identified in the genome of Qingke via HMM profiling. Of them, 25 TLPs contained a signal peptide at the N-terminus, with most proteins predicted to localize in the cytoplasm or outer membrane. Sequence alignment and motif analysis revealed that the five REDDD residues required for β-1,3-glucanase activity were conserved in 21 of the 36 Qingke TLPs. Phylogenetically, the TLPs in plants are clustered in 10 major groups. Our analysis of gene structure did not detect an intron in 15 Qingke *TLPs* whereas the other 21 did contain 1–7 introns. A diverse set of *cis*-acting motifs were found in the promoters of the 36 *TLPs*, including elements related to light, hormone, and stress responses, growth and development, circadian control, and binding sites of transcription factors, thus suggesting a multifaceted role of TLPs in Qingke. Expression analyses revealed the potential involvement of *TLPs* in plant defense against biotic and abiotic stresses. Taken together, the findings of this study deepen our understanding of the *TLP* family genes in Qingke, a staple food item in Tibet, which could strengthen future investigations of protein function in barley and its improved genetic engineering.

## Introduction

Due to their sessile nature, plants have evolved a sophisticated immune system to overcome challenges from potential pathogenic microorganisms. Current immune theory argues for a two-tiered system for how plants engage in a defense response against pathological invaders, in which plant innate immunity is defined as a two-layered model consisting of pattern-triggered immunity (PTI) and effector-triggered immunity (ETI) (Jones and Dangl, 2006). As the primary barrier of plant defense, activation of PTI triggers the early response of host’s immune system, resulting in a burst of ROS (reactive oxygen species) production and the coordinated expression of defense-related genes (Jwa and Hwang, 2017). However, virulent pathogens could breach the defense of the PTI layer and thereby initiate ETI-level immunity, which entails a stronger and more sustained response to invading pathogens (Yuan et al., 2021b). Recent findings have revealed that pattern-recognition receptors are also required for NLR-mediated ETI, highlighting the interaction between the PTI and ETI modules (Ngou et al., 2021; Yuan et al., 2021a). Activating PTI and ETI often releases signaling molecules from the site of infection, and thus potentiate resistance against incoming pathological threats in distal uninfected tissues (Durrant and Dong, 2004). This resistance mechanism, which is accompanied by the expression and accumulation of pathogenesis-related (PR) proteins, is termed systematic acquired resistance (SAR).

Thaumatin-like proteins (TLPs), also known as the PR-5 family protein, are so named because of their high similarity to thaumatin, a sweet-tasting protein originally isolated from *Thaumatococcus danielli* (Wel and Loeve, 1972). The existence of TLPs has since been confirmed in a diverse range of species, including those of fungi, animals, and plants. Not surprisingly, the functions of TLP proteins are also diversified and their involvement in various processes has been demonstrated. For example, in fungi, homologues of TLP can exhibit β-1,3-glucanase activity, playing active roles in cell wall degradation and spore diffusion of the fruiting body (Sakamoto et al., 2006). Through structural modeling, animal TLPs were also found to contain the structure responsible for putative glucanase activity (Brandazza et al., 2004). The existence of TLPs has been confirmed in the secretome of the pine wood nematode, which could facilitate its parasitism by inducing hypersensitive responses in the host tree (Kirino et al., 2020). In plants, TLPs have been detected in a wide variety of species, ranging from algae to angiosperms (Shatters et al., 2006). Recent report of TLPs in wheat confirmed their involvement in development and defense, and recombinant expression of TaTLP2-B conferred enhanced tolerance to salt, cold, osmotic and heat stresses for *Saccharomyces cerevisiae* (Sharma et al., 2022).

Typical TLPs share common structural features of the PR-5 family, possessing an acidic cleft that enables their binding of β-1,3-glucan (Grenier et al., 1999). This cleft, formed by five well-conserved amino acid residues—one glutamic acid, one arginine, and three aspartic acid residues (REDDD)—is the crucial structure for conferring antifungal activity. When grouped by their molecular weight (MW), TLPs may be categorized into two types: large and small (Liu et al., 2020). For large TLPs, their MW spans 21 to 26 kDa, and they harbor 16 cysteine residues paired into eight disulfide bonds. Small TLPs have a MW of 16–17 kDa, containing 10 cysteine residues forming five disulfide bridges. These intramolecular disulfide bonds stabilize the structure of TLPs, enabling them to endure unfavorable conditions (such as extreme pH levels and high temperatures) and resist protease degradation (Sharma et al., 2022).

Thaumatin-like proteins are associated with plant defense responses against both biotic and abiotic stresses. The transcription of *TLP* genes can be induced significantly after exposure to drought (Misra et al., 2016), freezing (Kuwabara et al., 2002), and salinity (Su et al., 2021) treatments, and transgenic lines overexpressing *TLP* genes could simultaneously exhibit resistance to biotic and abiotic stresses (Chowdhury et al., 2017). When expressed *in vitro*, TLPs inhibited the growth of fungal pathogens by lysing spores, impeding spore germination, and hindering hyphal growth (Abad et al., 1996; Rajam et al., 2007). In barley plants, HvTLP8, a malting-quality-associated QTL (quantitative trait locus), was implicated in β-glucan transformation in a redox-dependent manner (Singh et al., 2017).

Tibetan hulless barley (*Hordeum vulgare* L. var. *nudum*), known as “Qingke” in Chinese, is the only crop plant capable of a yield in regions lying above 4500 m elevation under natural conditions (Lin et al., 2018). As the staple food of Tibetanese, Qingke is tightly linked to food security and feed production in high-elevation areas. Recent developments in sequencing technology have propelled gene analysis forward, making it now feasible to comprehensively analyze a gene family using its transcriptomic or genomic sequencing data (Wang et al., 2021). In the present study, we carried out a comprehensive analysis of the *TLP* family genes in Qingke, by considering their pleiotropic role. Our study uncovered the genetic characteristics of *TLPs* in Qingke, and revealed the potential roles they play in this barley’s defense against pathogenic infections and drought and cold stresses.

## Materials and methods

### Identification and analysis *Thaumatin-like proteins* genes from Qingke

The genomic data of Tibetan hulless barley was downloaded from the Qingke genome database^1^ (Zeng et al., 2020). Preliminary identification of Qingke *TLP* sequences was performed by a Hidden Markov Model (HMM) search using the HMMER 3.0 program (Finn et al., 2011), for which the thaumatin domain (PF00314) served as the query term and a predefined *E*-value cutoff of 1e^–5^ was used. Positive hits were further aligned using the BioEdit tool to remove any redundancies. Sequences were then submitted to Pfam^2^, SMART^3^, and CCD^4^ databases for reconfirmation of the thaumatin domain (Mistry et al., 2020); only those possessing a complete thaumatin domain were classified as TLPs in the Qingke genome. Theoretical computation of the molecular weight (MW) and isoelectric point (pI) was done using the ProtParam tool^5^. To predict the signal peptides and sites of cleavage, the sequences were submitted to the SignaIP 5.0 server^6^ (Almagro Armenteros et al., 2019), while the proteins’ subcellular localization was determined with Plant-mPLoc (Wu et al., 2011) and Plant-mSubP (Sahu et al., 2019).

### Gene structure and motif analysis

Conserved motifs in the Qingke TLPs were identified using the MEME (Multiple Em for Motif Elicitation) suite v5.4.1^7^ (Bailey et al., 2015). The following parameters were applied for motif search: number of repetitions, any; maximum number of motifs, 10; and the minimum and the maximum motif length were configured as 6 and 100 residues respectively. To analyze and visualize the gene structure of *TLP*s, their CDS sequences and corresponding genomic fragments were uploaded to the Gene Structure Display Server^8^ (Hu et al., 2015).

### Phylogenetic analysis and homologous modeling of Qingke Thaumatin-like proteins

Sequence information of TLPs in the *Arabidopsis thaliana*, *Cucumis melo*, *Hordeum vulgare*, *Oryza sativa*, and*Brachypodium distachyon* genomes were obtained from previous reports (Iqbal et al., 2020; Liu et al., 2020; Zhang et al., 2021; Sharma et al., 2022). The phylogenic relationships among TLPs of the six species was inferred from a tree built in MEGA 11^9^ using the maximum likelihood (ML) method (Tamura et al., 2021). The constructed phylogeny was then visualized in EvolView^10^ (Subramanian et al., 2019). The three-dimensional structure of each TLP was predicted via homologous modeling, using the 3D structure of the cherry allergen Pru av 2 protein (2ahn.1.A) as a template (Huet et al., 2013).

### Prediction of *cis*-elements in the promoter region of Qingke *Thaumatin-like proteins*

For each *TLP* gene, the sequence of a genomic fragment located 2 kb upstream of the ATG start codon was extracted, and this used as a query for motif prediction. The *cis*-elements present in the promoter region were predicted by uploading the sequences to the PlantCARE server^11^ (Lescot et al., 2002). To visualize the *cis*-elements, the Gene Structure Display Server was used.

### Expression analysis of Thaumatin-like protein genes in response to biotic and abiotic stresses

The raw data from three published studies (Liang et al., 2017; Yuan et al., 2017, 2018) were downloaded from the Sequence Read Archive (SRA), and used for the expression analysis of Qingke *TLP* genes in response to powdery mildew infection and drought and cold stresses. Detailed information of this transcriptomic data appears in Supplementary Table 1. Data analysis was done using the pipeline provided by TBtools (Chen et al., 2020). First, any sequencing adaptors were filtered out using Trimmomatic (Bolger et al., 2014), then the resulting clean data was aligned to the Qingke reference genome by Hisat2 (Kim et al., 2019), and finally quantified by StringTie (Pertea et al., 2015). After transforming them logarithmically, the transcripts per million (TPM) data were used to compare the transcription levels of the *TLP* genes in response to the different treatments.

### Plant materials, RNA isolation, and qRT-PCR

Seeds of the Tibetan hulless barley variety ‘Dulihuang’ were used as the material for the qPCR. Seeds were first sterilized with 10% bleach for 5 min, then rinsed thrice with deionized water. Next, soaked seeds were transferred to Petri dishes filled with wet filter papers and allowed to incubate for 48 h at 22°C to germinate. Sprouted seeds were planted into peat moss-containing pots (9.5 cm in diameter), and cultivated under controlled conditions (at 55% relative humidity and 22°C under a 16-h/8-h light/dark photoperiod with a light intensity of 20 000 lx) in growth chambers. For their treatment with phytohormones, seedlings at the 4-leaf stage were subjected to foliar spraying of a methyl jasmonate (20 mM) or sodium salicylate (10 mM) solution. Leaves were sampled at 0, 2, 6, 8, 12 and 24 h post-treatment, and frozen immediately in liquid nitrogen and kept at –80°C until used. To isolate the total RNA, the RNAprep Pure Plant Kit (TIANGEN, China) was used according to its manufacturer’s instructions. The extracted RNA was first treated with DNase I to eliminate possible contamination by DNA, and all samples then subjected to 1% agarose gel electrophoresis to check their integrity. The concentration of total RNA isolated from a given sample was determined using a NanoDrop spectrophotometer. To quantify the transcript levels of Qingke TLP genes by qPCR, total RNA was reverse-transcribed into cDNA, using the HiScript III RT SuperMix for qPCR kit (+ gDNA wiper) (Vazyme Biotech, Nanjing, China). Then, using the AceQ Universal SYBR qPCR Master Mix (Vazyme Biotech, Nanjing, China), the qPCR’s 20-μL reaction mixture was prepared for each test. Thermal cycling reactions were run in the MX3000P qPCR thermo-cycler (Stratagene, United States). The Qingke *HSP* gene served as the internal reference (Cai et al., 2018). The relative expression software tool (REST) 2009 was used to derive the relative expression levels of genes (Pfaffl et al., 2002). Detailed information of the primers used can be found in Supplementary Table 2.

## Results

### Genome-wide identification of *Thaumatin-like proteins* in Qingke

To identify TLPs in the Qingke genome, we performed a thorough search using the HMM profile of the thaumatin domain (PF00314) as a query. After redundancy removal, candidate sequences were submitted to the SMART, CCD, and Pfam databases to reconfirm the thaumatin domain, and a total of 36 Qingke TLPs thus identified. Details about their physical and biochemical properties are presented in Table 1. Consisting of 153 to 728 amino acids, Qingke TLPs had molecular weights (MW) ranging from 16.21 to 79.97 kDa. According to their calculated theoretical isoelectric point, 12 of the 36 TLPs were basic while the other 24 were acidic; GRAVY values were below 0 for 17 TLPs, implying these proteins’ strong hydrophilicity whereas they were greater than 0 for the other 19 TLPs, indicating their hydrophobicity; and 25 TLPs harbored a signal peptide at the N-terminus, whose length spanned 17 to 34 amino acids. Details concerning their sites of cleavage are in Table 1. We predicted the subcellular localization of all 36 TLPs. These results showed that most of these proteins localized to the extracellular or periplasmic spaces, with only four localizing to the cytoplasm or outer membrane.

**TABLE 1:** Physical and biochemical properties of the TLPs identified in Qingke.

Sequence ID	Length (aa)	MW (kD)	pI	SP	SL	GRAVY
HOVUSG4889300	330	34.17027	5.19	31-32	Extracellular	–0.085
HOVUSG1548800	325	32.66853	4.64	–	Extracellular	0.098
HOVUSG2056400	320	32.03172	4.51	30-31	Extracellular	0.204
HOVUSG2715500	324	32.24737	5.03	24-25	Extracellular	0.306
HOVUSG2403700	356	35.39969	4.80	–	Extracellular	0.034
HOVUSG5777800	360	37.07682	5.93	24-25	Extracellular	0.034
HOVUSG5063300	438	45.90470	6.23	19-20	Extracellular	–0.068
HOVUSG3013000	248	25.42904	8.78	23-24	Extracellular	0.109
HOVUSG2714800	360	37.56808	5.48	–	Extracellular	–0.098
HOVUSG3359300	259	25.74488	4.79	20-21	Extracellular	0.241
HOVUSG6064000	258	27.09720	8.43	23-24	Periplasmic	0.267
HOVUSG2056300	340	34.12019	4.76	27-28	Extracellular	0.188
HOVUSG5482900	264	27.80845	6.68	–	Extracellular	–0.124
HOVUSG1461300	352	35.36611	4.71	–	Extracellular	–0.198
HOVUSG6545900	571	61.86448	5.63	–	Cytoplasmic	0.001
HOVUSG6188700	261	26.08608	5.18	34-35	Extracellular	0.207
HOVUSG1336900	316	33.50597	7.30	–	Extracellular	0.045
HOVUSG3726900	279	27.80427	6.83	–	Extracellular	0.259
HOVUSG6245400	250	26.03168	7.47	24-25	Periplasmic	0.044
HOVUSG6558000	227	23.64379	7.36	24-25	Periplasmic	–0.030
HOVUSG5063500	226	23.72595	7.33	24-25	Periplasmic	–0.022
HOVUSG5062800	472	51.36320	5.47	–	Periplasmic	–0.203
HOVUSG3036500	728	79.96609	6.82	24-25	OuterMembrane	–0.237
HOVUSG0169900	563	61.27101	7.44	18-19	Periplasmic	–0.129
HOVUSG3036300	641	71.00618	6.38	28-29	Cytoplasmic	–0.224
HOVUSG0170500	527	57.91669	5.95	–	Cytoplasmic	–0.297
HOVUSG0170200	572	63.75936	7.94	30-31	Cytoplasmic	–0.177
HOVUSG3065300	173	17.54947	4.51	20-21	Extracellular	0.050
HOVUSG5062900	268	27.92257	7.50	22-23	Periplasmic	–0.030
HOVUSG3036800	427	46.47299	8.13	–	Periplasmic	–0.253
HOVUSG5063600	171	17.82000	6.52	22-23	Extracellular	–0.019
HOVUSG6418000	184	18.35486	8.02	26-27	Periplasmic	0.149
HOVUSG5872500	229	23.81215	4.90	21-22	Periplasmic	0.121
HOVUSG4917800	240	24.63917	7.52	27-28	Periplasmic	0.129
HOVUSG2829200	175	18.04821	5.02	22-23	Extracellular	0.112
HOVUSG5063200	153	16.21212	6.52	17-18	Extracellular	–0.039

MW, molecular weight; pI, isoelectric point; SP: Signal peptide cleavage site; SL: Subcellular localization; GRAVY, grand average of hydropathicity.

### Sequence alignment and phylogenetic analysis of Qingke Thaumatin-like proteins

Multiple alignments of Qingke TLPs’ sequences confirmed the conservatism of their feature structures. Cysteines are considered crucial for structure formation and protein stability of TLPs. A total of 16 cysteines were found highly conserved in 22 of the 36 Qingke TLPs (Supplementary Figure 1). For the remaining 14 proteins, intra-molecular disulfide bridges could still be formed, though the number and location of the cysteine residues changed. The five conserved REDDD residues formed the acidic cleft responsible for the β-1,3-glucanase activity of TLPs. The complete set of these five residues was detected in 21 Qingke TLPs. This meant that for the other 15 TLPs their glucanase activity might be impaired or even gone. A conserved CQTGDCGG motif, it involved in β-glucan binding in a redox-dependent manner, was observed in six TLPs (HOVUSG6418000, HOVUSG3065300, HOVUSG0170500, HOVUSG0169900, HOVUSG3036500, and HOVUSG1461300).

**FIGURE 1:**
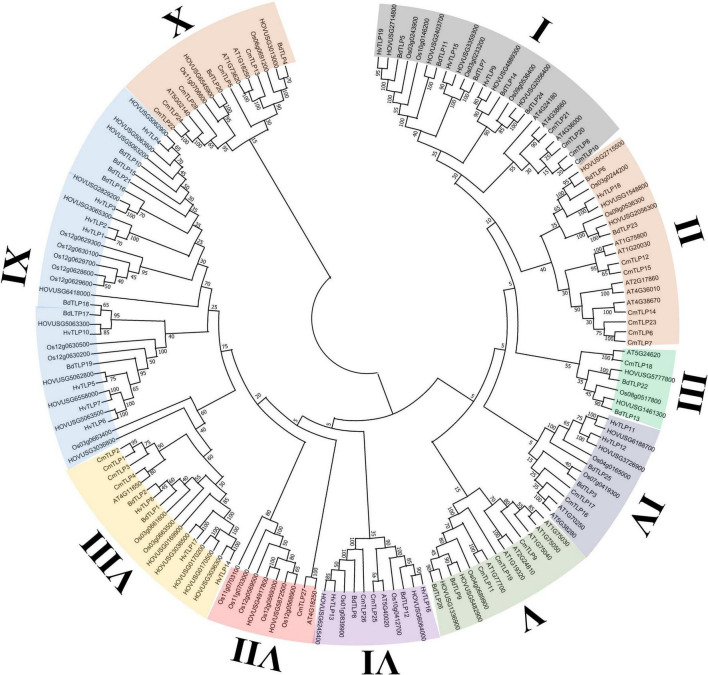
Phylogenetic relationships of TLPs from Qingke, *Hordeum vulgare*, *Cucumis melo*, *Oryza sativa*, *Brachypodium distachyon*, and *Arabidopsis thaliana*. The phylogeny was constructed by MEGA 11, using the maximum likelihood (ML) method with 1000 bootstrap replicates. The inferred phylogeny was visualized in the Evolview online server. TLPs were classified into 10 groups, these indicated by different background colors.

The evolutionary relationships among TLPs were explored via phylogenic inference with homologs from Qingke, *Hordeum vulgare*, *Cucumis melo*, *Oryza sativa*, *Brachypodium distachyon*, and *Arabidopsis thaliana*. TLPs of these three species were classified into 10 major groups, with each clade containing at least two TLP from Qingke (Figure 1). Clade IX was the largest group identified, comprising 11 Qingke TLPs, 8 HvTLPs, 8 OsTLPs and 7 BdTLPs. By contrast, clade III was the smallest, formed by one OsTLP, one AtTLP, One CmTLP, two BdTLPs and two Qingke TLPs. The 21 Qingke TLPs containing the five complete REDDD residues were mainly distributed in seven groups: clade I, clade II, clade III, and clade V. The TLPs from these groups tended to possess the β-1,3-glucanase activity; hence, they might be primary candidates involved in Qingke’s resistance to disease.

### Analysis of domain composition, motif pattern and gene structure of the Thaumatin-like proteins family in Qingke

The functional properties of proteins depend largely on domain composition. All the 36 identified Qingke TLPs possessed a characteristic thaumatin domain (with lengths ranged from 129 to 234 AAs), confirming their identity as TLPs (Supplementary Table 3). A protein kinase domain was detected in six TLPs (HOVUSG3036500, HOVUSG0169900, HOVUSG3036300, HOVUSG0170500 and HOVUSG0170200), demonstrating features of typical thaumatin-like protein kinases (TLPKs). A cyclin N-terminal domain was found in HOVUSG6545900, but whether this thaumatin-domain-containing protein is involved in cell cycle control still await to be verified.

**FIGURE 2:**
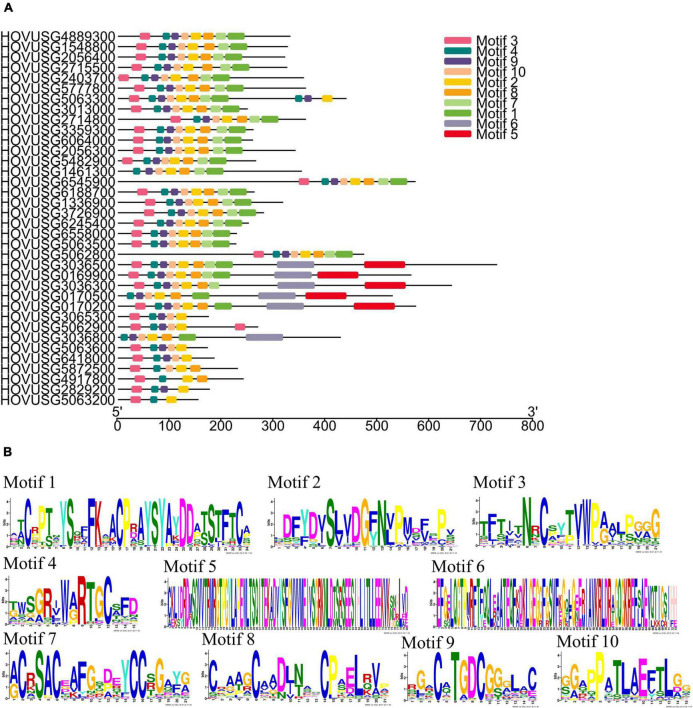
Conserved motifs identified in the Qingke TLP proteins. **(A)** Distribution of conserved motifs. **(B)** Sequence logo of motifs identified. The height of a symbol reflects the relative frequency of the amino acid at that position.

To identify and analyze the distribution of the conserved motifs, their corresponding TLP sequences were submitted to the MEME server. As Figure 2A shows, a total of 10 conserved motifs were detected in Qingke TLPs; details for these identified motifs appear in Figure 2B. Two motifs, motif 2 and motif 4, were highly conserved across the 36 Qingke TLPs. But for some motifs, such as motif 5 and motif 6, their presence was only detected in six TLPKs, in which these two motifs correspond to the conserved sequences of the protein kinase domain (Supplementary Figure 2). Concerning the other 6 motifs, they were found present in most of the TLP members. These results suggested the Qingke TLP family proteins shared highly conserved structural basis.

**FIGURE 3:**
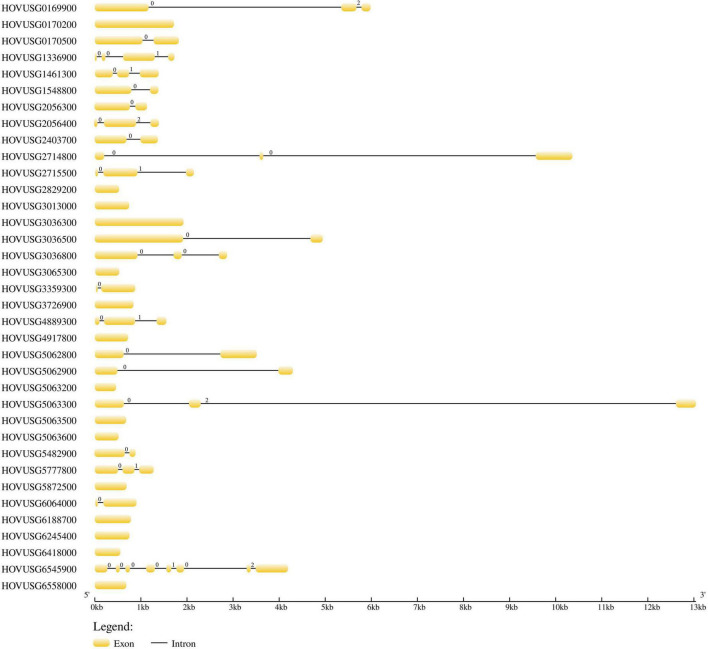
Exon–intron structure of the Qingke *TLP* genes. Yellow boxes and black lines respectively indicate the exons and introns. The numbers (0, 1, and 2) indicate phases of introns.

Gene structure analysis revealed that the number of introns varied from 0 to 7 in the Qingke *TLPs*. Of the 36 *TLP* genes, 15 contained no introns (Figure 3), and among the other 21 intron-containing genes, *HOVUSG6545900* had the most introns. Our results of exon-intron analysis echoed previous findings for melon and maize *TLPs*, for which a significant proportion of genes are intron-free. Analysis of intron phase revealed that the majority of the introns were phase 0 (28 out of 38), and phase-1 and phase-2 introns were 6 and 4, respectively.

### Tertiary structure of Thaumatin-like proteins in Qingke

**FIGURE 4:**
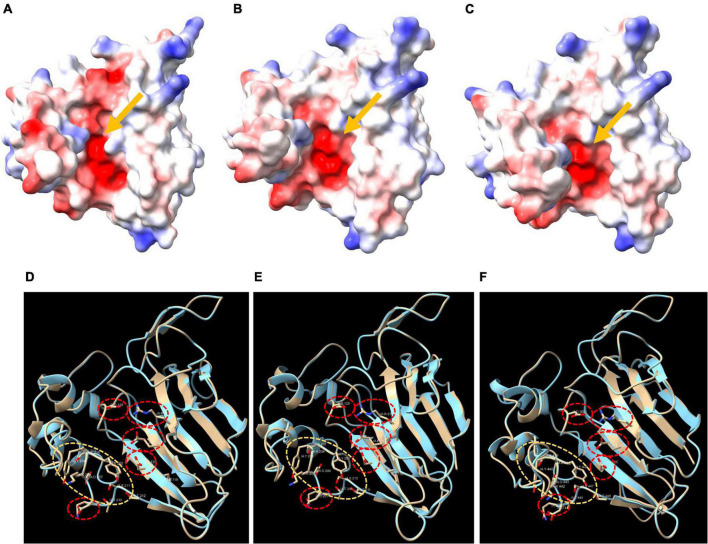
The electrostatic surface and the 3D structure of three Qingke TLPs. **(A–C)** The electrostatic surface of HOVUSG6558000, HOVUSG5063500 and HOVUSG5062800, respectively. Surfaces where the electrostatic potential is positive are colored blue, while those negatively charged are colored red. The arrows point to the acidic clefts formed by five conserved residues (REDDD). **(D–F)** The 3D structure of HOVUSG6558000, HOVUSG5063500 and HOVUSG5062800, with residues of the REDDD motif (red circles) and the bottom of acidic cleft forming regions (yellow circles) shown.

Typical TLPs harbored an acidic cleft structure formed by the five conserved REDDD residues. We generated the surface electrostatic potential and the 3D structure of three selected Qingke TLPs (Figure 4), and a typical acidic cleft was observed (indicated by the arrow), which were formed by the five REDDD residues (Figures 4D-F, red circle). Residues located at the bottom of the acidic cleft forming regions were also highly conserved (Figures 4D-F, yellow circle). Through structure modeling, we found that Qingke REDDD-containing TLPs probably possessed the antifungal traits of typical TLPs.

### *Cis*-elements in the promoters of Qingke *Thaumatin-like proteins* genes

**FIGURE 5:**
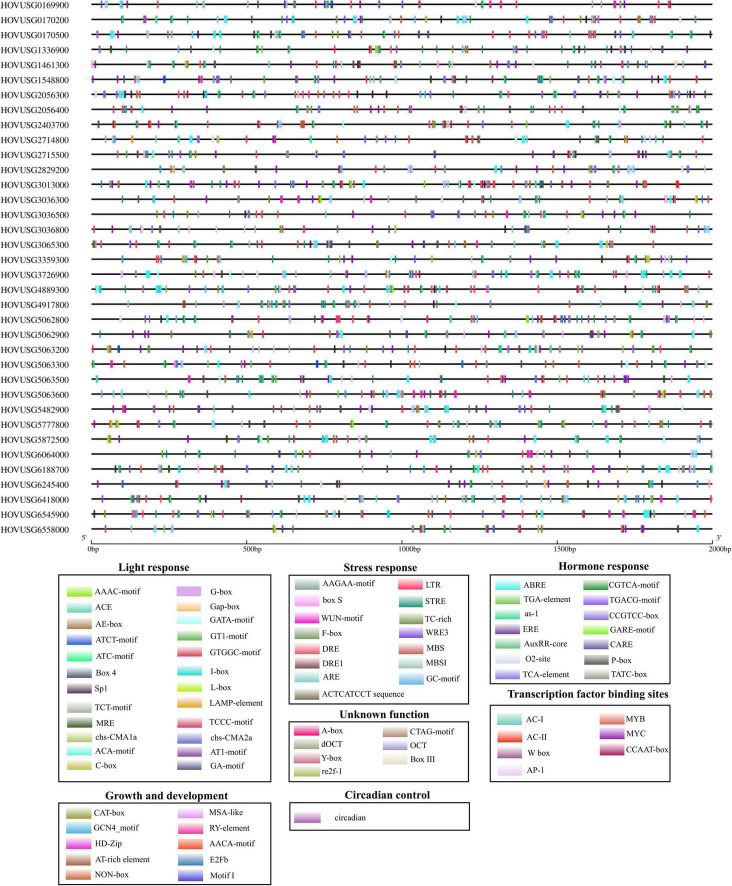
Distribution of *cis*-elements in the promoters of Qingke *TLP* genes. Black lines indicate the promoters. *Cis*-elements differing in function are color-coded accordingly.

To investigate the *cis*-acting motifs controlling *TLPs’* expression, the 2-kb promoter sequence of each of Qingke *TLP* was submitted to the PlantCARE database. For the 36 *TLPs*, a total of 2092 *cis*-regulatory elements were identified in their promoters, these belonging to 7 categories based on which biological processes they are involved in Figure 5. Hormone-responsive motifs were the most abundant type, constituting 34.08% of the total number of elements identified (Supplementary Figure 3). Motifs controlling ABA (ABRE), SA (as-1, TCA-element), MeJA (CGTCA-motif, TGACG-motif), and ET (ERE) responses were the major types of the hormone-responsive elements (Supplementary Figure 4B). *Cis*-elements identified as transcription factor binding sites were also significantly enriched, accounting for 17.78% of all motifs identified (Supplementary Figure 3). Overall, 22 types of light-responsive elements were detected, demonstrating high diversity in the motif composition. Also discernible were *cis*-elements that participate in the transcription regulation of plants’ stress responses, their growth and development processes, as well as circadian control (Supplementary Figure 3). The presence of these regulatory motifs in the promoter suggested that *TLPs*’ expression is influenced by a diverse set of regulatory factors.

### Expression profile of *Thaumatin-like proteins* in Qingke plant under biotic stress

**FIGURE 6:**
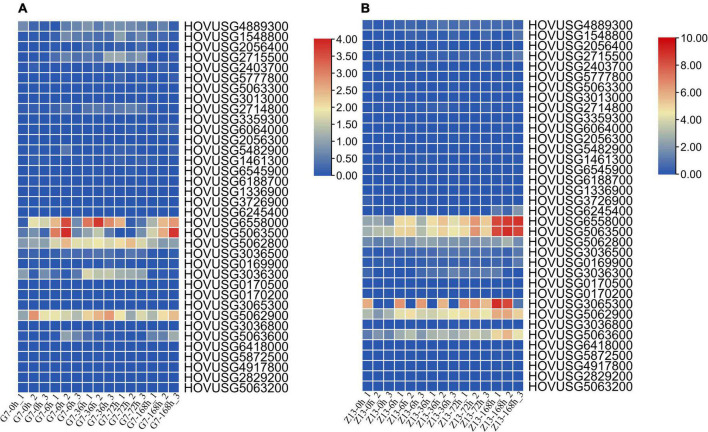
Expression of Qingke *TLP* genes in response to powdery mildew infection. Their expression levels at 0, 6, 36, 72, and 168 h post-inoculation (hpi) **(A)** in the powdery mildew-resistant cultivar Gannongda7 (G7) and **(B)** the powdery mildew-sensitive cultivar Zangqing13 (Z13). The RNA-seq data was downloaded from SRA database, and aligned to the Qingke genome to calculate the expression levels of *TLP* genes. Bars are colored according to the log2 (TPM + 1) values.

For the expression profiles of *TLPs* in response to biotic stress, the RNA-Seq data from a previous study was reanalyzed here. As seen in Figure 6, *HOVUSG6558000*, *HOVUSG5063500*, *HOVUSG5062800*, and *HOVUSG5062900* were distinguished as the major responsive genes to powdery mildews infection, in both the G7 resistant cultivar and the Z13 susceptible cultivar. However, these four genes were more rapidly induced in the G7 than Z13 cultivar, implying their possible role in *TLP*-mediated resistance. Expression profile results revealed that four other *TLPs*, namely *HOVUSG4889300*, *HOVUSG1548800*, *HOVUSG2715500*, and *HOVUSG3036300*, might also contribute importantly to plant defense in the G7 resistant cultivar. In the Z13 susceptible cultivar, only the expression of *HOVUSG3065300* and *HOVUSG5063600* was detected. By reanalyzing the transcriptomic data, we succeeded in identifying the major biotic stress-responsive *TLPs* in Qingke. With further functional analyses, these genes could prove useful for improving barley resistance traits via genetic engineering.

### Expression patterns of Qingke *Thaumatin-like proteins* under abiotic stress

**FIGURE 7:**
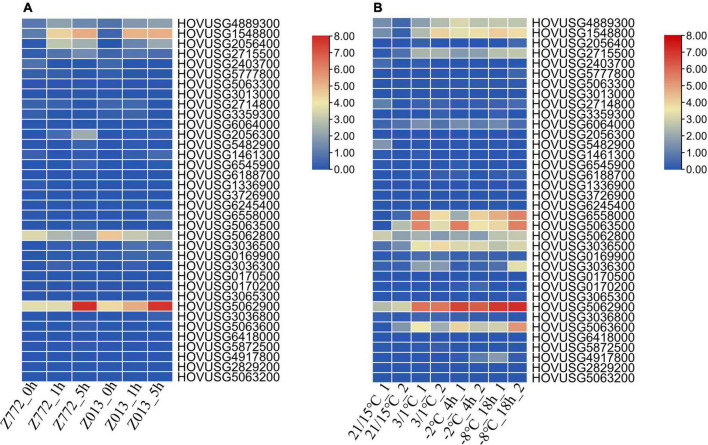
Expression of Qingke *TLP* genes in response to drought and cold stresses. **(A)** Expression of Qingke *TLP* genes in drought-tolerant cultivar Z772 and drought-sensitive cultivar Z013 at 0, 1, and 5 h post-stress treatment. **(B)** Expression of Qingke *TLP* genes in the cold-tolerant cultivar Tibet148 subjected to 20/15°C (day/night), 3/1°C (day/night), –2°C (for 4 h) and –8°C (for 18 h) stress treatments. The RNA-seq data was downloaded from SRA database, and aligned to the Qingke genome to calculate the expression levels of *TLP* genes. Bars are colored according to the log2 (TPM + 1) values.

Our reanalysis of the expression data indicated that most *TLPs* of Qingke were unresponsive to drought (Figure 7A). Drought did, however, induce the expression of *HOVUSG1548800*, *HOVUSG2056400*, and *HOVUSG5062900*, in the drought-tolerant Z772 cultivar as well as the drought-sensitive Z013 cultivar. But a lack of water exerted a negative impact on the transcription of *HOVUSG5062800*, whose expression is maintained at high level under normal conditions. At 5 h after the stress treatment, *HOVUSG2056300*’s expression was significantly upregulated in the drought-tolerant Z772. The drought-inducibility of *TLPs*, albeit a few, nonetheless suggested these genes figure prominently in drought adaption in Qingke.

With respect to cold stress, nine *TLPs*—*HOVUSG4889300, HOVUSG1548800, HOVUSG2715500, HOVUSG6558000, HOV USG5063500, HOVUSG5062800, HOVUSG3036500, HOV USG5062900*, and *HOVUSG5063600*—were identified as major responsive genes (Figure 7B). Their expression levels were strongly induced by cold stress, especially under the subzero –2°C and –8°C treatments. Some of these *TLPs* were also identified as major participants of powdery mildews infection. Our results suggested that members of the *TLP* family might play multiple roles in how Qingke’s defense response against biotic and abiotic stresses.

### Expression patterns of *Thaumatin-like proteins* under the hormone treatments

**FIGURE 8:**
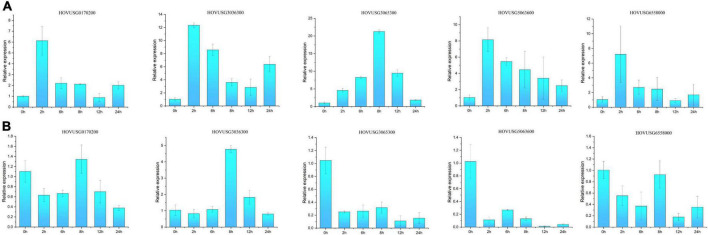
Relative expression levels of *TLP* genes responding to sodium salicylate (SA) and methyl jasmonate (MeJA) treatments. **(A)** Expression patterns of *HOVUSG6558000*, *HOVUSG3036300*, *HOVUSG0170200*, *HOVUSG3065300* and *HOVUSG5063600* in response to the SA treatment. **(B)** Expression patterns of *HOVUSG6558000*, *HOVUSG3036300*, *HOVUSG0170200*, *HOVUSG3065300* and *HOVUSG5063600* in response to the MeJA treatment. The expression level of the untreated control (0 h) was used to calculate the relative expression levels at different time-points after imposing the hormone treatments. Bars are the mean ± SD of expression levels obtained from three independent biological replicates.

Salicylic acid (SA) and jasmonic acid (JA) exert direct but antagonistic effects on the expression of some *TLP* genes, suggesting differential roles of TLPs in the defense response against biotrophic vis-à-vis necrotrophic pathogens. Five Qingke *TLPs* were selected to characterize their expression patterns in response to SA and JA treatments. As illustrated in Figure 8, most of the selected TLPs showed strong and acute induction by SA, especially *HOVUSG6558000, HOVUSG3036300, HOVUSG0170200*, and *HOVUSG5063600*, all of which peaked at 2 h post-treatment. Transcription of *HOVUSG3065300* also exhibited strong inducibility, in that its expression level reached 21.26 times of the untreated control (0 h) at 8 h after the SA treatment. Acting antagonistically, MeJA exerted negative effects on the expression of most Qingke *TLPs*, such as *HOVUSG3065300*, *HOVUSG5063600*, *HOVUSG6558000* and *HOVUSG0170200*. Intriguingly, *HOVUSG3036300* was the only *TLP* induced by both MeJA and SA, suggesting it could have dual roles in SA- and JA-mediated defense signaling. Altogether, these results indicated that Qingke TLPs are active in the SA pathway, but their inducibility (i.e., sensitivity and intensity) is differentiated.

## Discussion

Thaumatin-like proteins (TLPs) are pivotal components of plant defense against both biotic and abiotic stresses. Genome-wide identification has verified the widespread presence of TLPs in a variety of fungal, plants, and animal species (Liu et al., 2010). Considering the profound roles of TLPs in plants’ defense and development (Munis et al., 2010), a comprehensive analysis conducted at the genome-wide scale would advance research on Qingke in several respects. Qingke has been relied upon as a major food source by Tibetans, having been domesticated from wild barley relatives some 4,500 to 3,500 years ago according to its extensive genome sequencing (Zeng et al., 2018). In the present study, we performed a genome-wide identification of *TLP* genes in Qingke, and analyzed their expression in response to biotic and abiotic stresses.

HMM profiling of thaumatin-domain-containing proteins facilitated our identification of 36 TLPs in Qingke. Most of the TLPs contained a signal peptide (SP) at the N-terminus, and were predicted to locate extracellularly (Faillace et al., 2019). In a previous study, Irfan et al. (2020) identified a total of 19 TLPs in the genome of barley, of which four HvTLPs contained the conserved CQTGDCGG motif. Expansion of the TLP family protein, as well as the CQTGDCGG-containing TLPs, was evident in Qingke. This suggests the TLP family has expanded significantly during the domestication and evolution process, despite differing identification procedures and cut-off values having been applied. The carbohydrate-binding motif (CQTGDCGG) of HvTLP8 binds to β-glucan in a redox-dependent manner, thereby altering the transformation of β-glucan during the germination and malting process (Singh et al., 2017). It is highly likely that the six CQTGDCGG-containing TLPs—HOVUSG6418000, HOVUSG3065300, HOVUSG0170500, HOVUSG0169900, HOVUSG3036500, and HOVUSG1461300—also have the β-glucan binding ability, and could regulate β-glucan transformation in Qingke in a similar way.

Cysteines are recognized for being crucial to the structure formation and protein stability of TLPs. Sequence alignments revealed that 22 of the 36 Qingke TLPs were highly conserved at 16 disulfide bond-formation cysteine residues (Supplementary Figure 1). The intra-molecular disulfide bridges formed by cysteines are known to be critical for not only TLPs but also for proteins of other PRs (such as PR1 and PR4) (Wang et al., 2017; Zhang et al., 2022). These cysteines augment the stability of PRs, especially when challenged by unfavorable conditions such as an extreme pH or protease degradation (Ghosh and Chakrabarti, 2008).

The anti-fungal property of TLPs is mainly ascribed to their glucanase activity. For most of the TLPs, the acidic cleft, a conserved structure formed by the REDDD motif, is responsible for binding of the β-1,3-glucan substrate (Liu et al., 2010). But the REDDD motif was not consistently conserved in all the TLPs (Faillace et al., 2019), hinting at differences in substrate binding affinity and antifungal activity. What makes the situation more complicated is that some TLPs possessing the electronegative acidic cleft are devoid of an antifungal property (Menu-Bouaouiche et al., 2003). In fruit plants such as banana (Xiao et al., 2019), grape (Tattersall et al., 1997), and cherry (Fils-Lycaon et al., 1996), an accumulation of TLPs was related to the softening of pulp or flesh. Some fruit TLPs, though with moderate β-1,3-glucanase activity, were found incapable of antifungal activity (Menu-Bouaouiche et al., 2003). Considering the complexity and the multifaceted role of TLPs in plant defense and development, further research is warranted to elaborate their function in Qingke.

The expression of *TLPs* can respond to a variety of stimuli, including salt, drought (Su et al., 2021), SA, MeJA, ABA, and ethephon (Singh et al., 2013), as well as host infections from a pathogenic virus (Cornelissen et al., 1986) or fungus (Wang et al., 2010). The fact that *TLPs*’ expression was inducible by multiple signals suggests their respective transcription is under the control of a diverse set of *cis*-regulatory elements. Sequence querying confirmed that the motifs regulating the transcription of Qingke *TLPs* were divergent in composition, with *cis*-elements involved in the hormone response being the most abundant type found. Motifs controlling ABA (ABRE), SA (as-1, TCA-element), ET (ERE), and MeJA (CGTCA-motif, TGACG-motif) responses were frequently identified in promoters of Qingke *TLPs*. Being among those genes recognized as a marker for SA-mediated systematic acquired resistance (SAR), the expression of *TLPs* exhibited strong induction when treated with exogenous SA (Ali et al., 2017). Existence of the as-1 and TCA-element in Qingke *TLP* promoters provides recognition sites for SA-mediated transcription regulation, implying the involvement of *TLPs* in this plant’s SA-mediated defense response. Given these findings, it is reasonable to conclude that *TLPs* function in the stress response and phytohormone-mediated signal transduction in Qingke. We also found *cis*-acting motifs involved in light response, stress response, growth and development processes, and circadian control; this strongly suggests the transcription of *TLPs* was concomitantly regulated by multiple stimuli.

Reanalyzing the RNA-seq data enabled us to examine the expression profiling of *TLPs* in response to biotic and abiotic stresses. Four *TLPs* (*HOVUSG6558000*, *HOVUSG5063500*, *HOVUSG5062800* and *HOVUSG5062900*), whose expression was significantly upregulated in both the G7 resistant cultivar and the Z13 susceptible cultivar, were identified as the major powdery mildew-responsive genes of the *TLP* family in Qingke. However, a more rapid induction of these four genes was observed in the G7 resistant cultivar, highlighting the involvement of *TLPs* in disease resistance. Further, the expression levels of *TLPs* were differentially regulated, depending on the stressing factor incurred. In *Rosa chinensis*, seven *RcTLPs* exhibited greater expression when exposed to drought or salinity stress. Three *RcTLPs* showed specific expression in leaves infected with *Botrytis cinerea* for 48 h (Su et al., 2021). In wheat, 50 *TaTLPs* were differentially expressed when exposed to *Blumeria graminis* or *Puccinia striiformis* infection (Sharma et al., 2022). More *TaTLPs* are characterized by responsiveness to abiotic stimuli, such as salt, heat, and osmotic stresses. Here, we found that different members of *TLP* family genes were differentially regulated when subjected to differing stresses. Our study unraveled the genetic characteristics of *TLP* family genes in Qingke, and helped to distinguish the core participants functioning in this plant’s mitigation of stressful conditions.

## Conclusion

**FIGURE 9:**
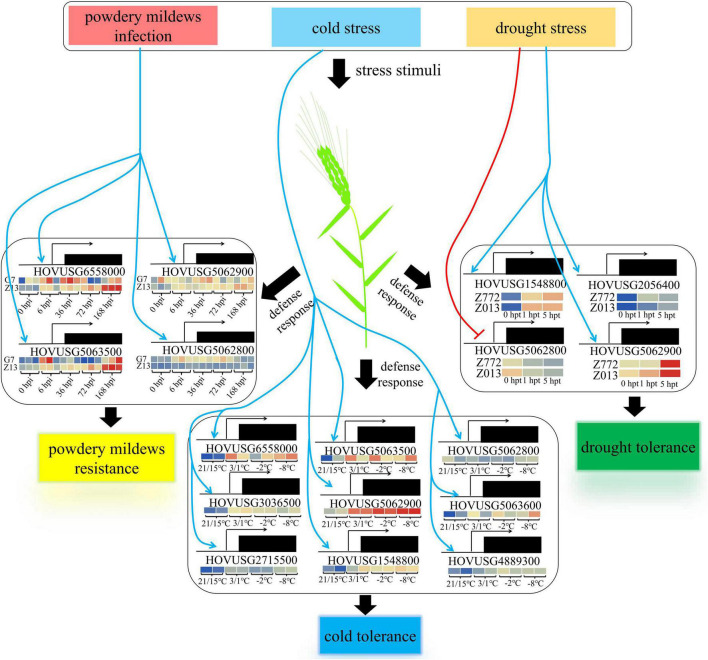
Proposed working model for *TLP*-mediated plant defense response in Qingke. Blue arrows indicate the promotion of gene transcription, while the red arrow indicates its negative regulation.

A total of 36 *TLPs* were identified from the Qingke genome via HMM profiling, and our analyses of gene expression and bioinformatic properties shed light on the functioning of TLP in plant defenses against biotic and abiotic stresses (Figure 9). Four *TLPs*, all exhibiting strong inducibility to powdery mildews infection, were identified as major members of the *PR-5* family participating in the plant’s response to biotic stress. Yet more *TLPs* were found actively involved in mitigating abiotic stresses, especially cold. In systemically characterizing the genetic features of the *TLP* family gene, we anticipate our findings will spur and inform subsequent investigations into protein functions and enzymatic properties of barley and closely related plant species.

## Data availability statement

The original contributions presented in the study are included in the article/Supplementary material, further inquiries can be directed to the corresponding authors.

## Author contributions

LeW, JL, and LiW designed and performed the experiments. ZX, WY, and KX devised the experiments. SW, QS, and WS helped with the data analysis and writing of the manuscript. All authors contributed to the article and approved the submitted version.

## Footnotes

1
http://show.genebang.com/project/index?n=barley

2
http://pfam.xfam.org/

3
http://smart.embl-heidelberg.de/

4
https://www.ncbi.nlm.nih.gov/cdd/

5
https://web.expasy.org/protparam/

6
https://services.healthtech.dtu.dk/service.php?SignalP-5.0

7
https://meme-suite.org/meme/tools/meme

8
http://gsds.cbi.pku.edu.cn

9
http://www.megasoftware.net

10
https://www.evolgenius.info/evolview/

11
http://bioinformatics.psb.ugent.be/webtools/plantcare/html/
