# Supplementary figures and images for: Genome-wide analysis of the Thaumatin-like gene family in Qingke (Hordeum vulgare L. var. nudum) uncovers candidates involved in plant defense against biotic and abiotic stresses
Source: Front Plant Sci. 2022 Aug 17;13:912296. doi: 10.3389/fpls.2022.912296 (PMC9428612; doi:10.3389/fpls.2022.912296)

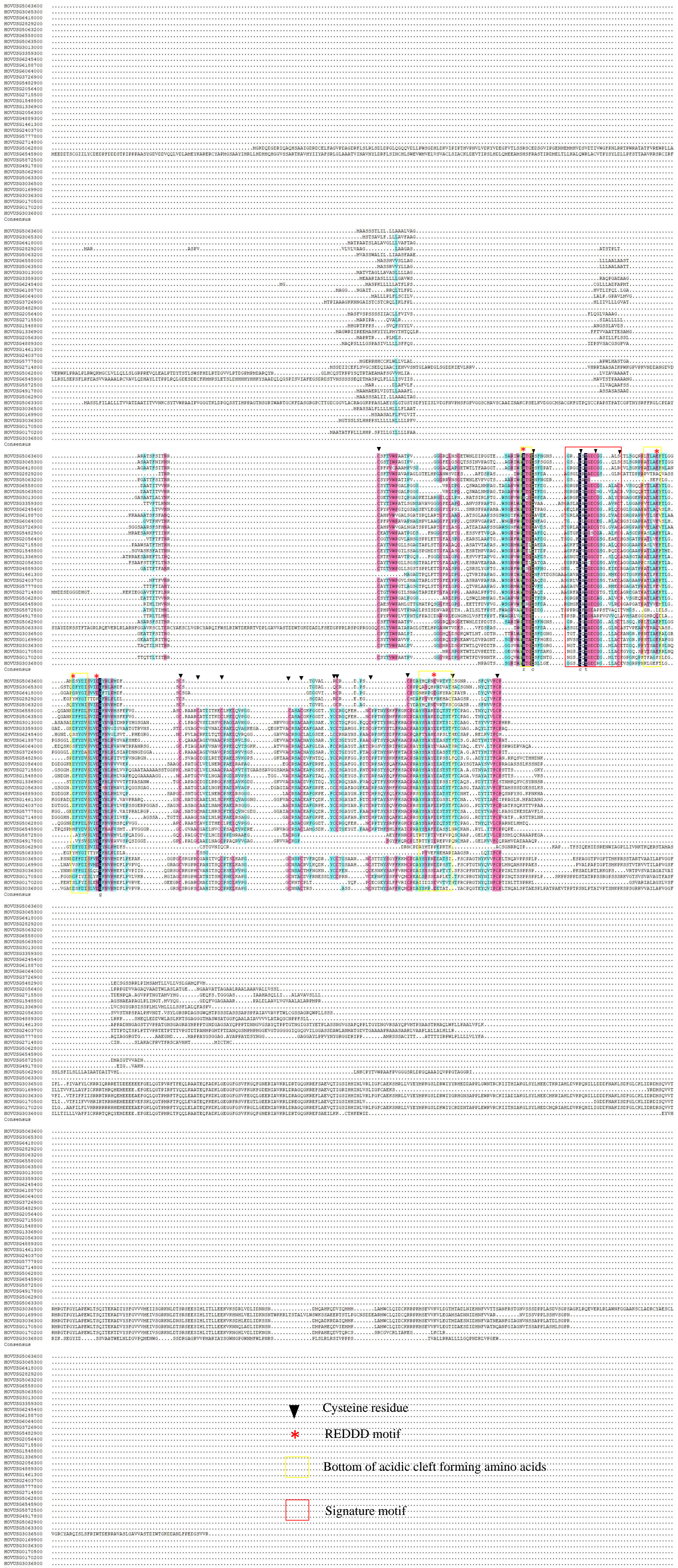

Supplement: Supplementary file 1 [file Data_Sheet_1.PDF]
