# Supplementary material for: Genome-wide analysis of the Thaumatin-like gene family in Qingke (Hordeum vulgare L. var. nudum) uncovers candidates involved in plant defense against biotic and abiotic stresses
Source: Front Plant Sci. 2022 Aug 17;13:912296. doi: 10.3389/fpls.2022.912296 (PMC9428612; doi:10.3389/fpls.2022.912296)
Supplement: Supplementary file 2 [file Data_Sheet_2.docx]

Supplementary Material

## Supplementary Figures


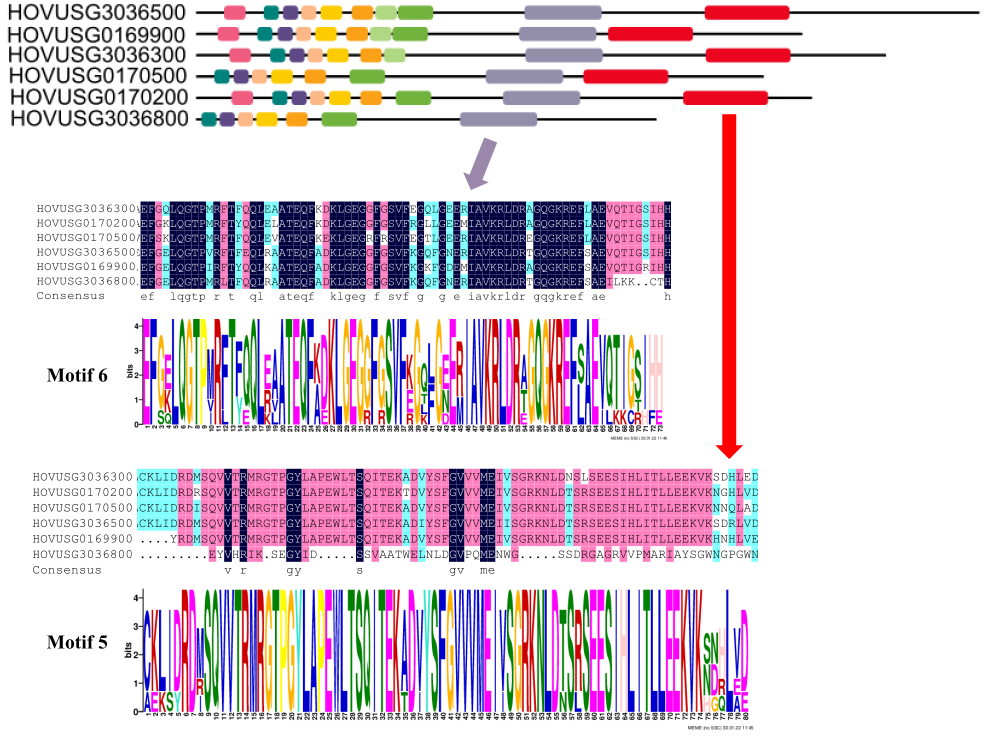


**Figure S2** Motif pattern and corresponding relations of motif 5 and motif 6 to protein sequences of six TLP kinases identified.

**
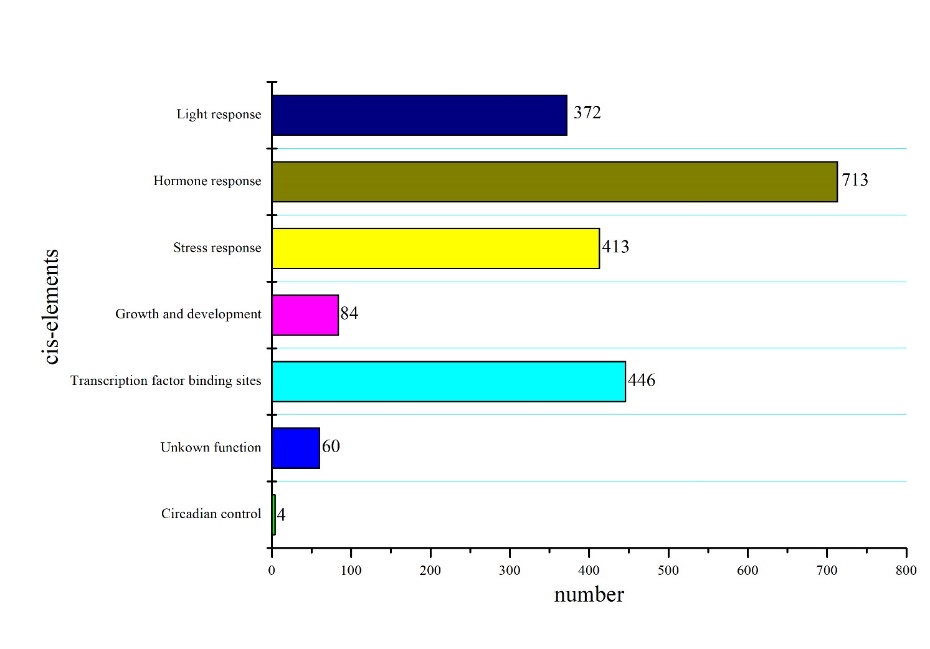
**

**Figure S3** Total number of cis-elements detected in each of the even categories.

**
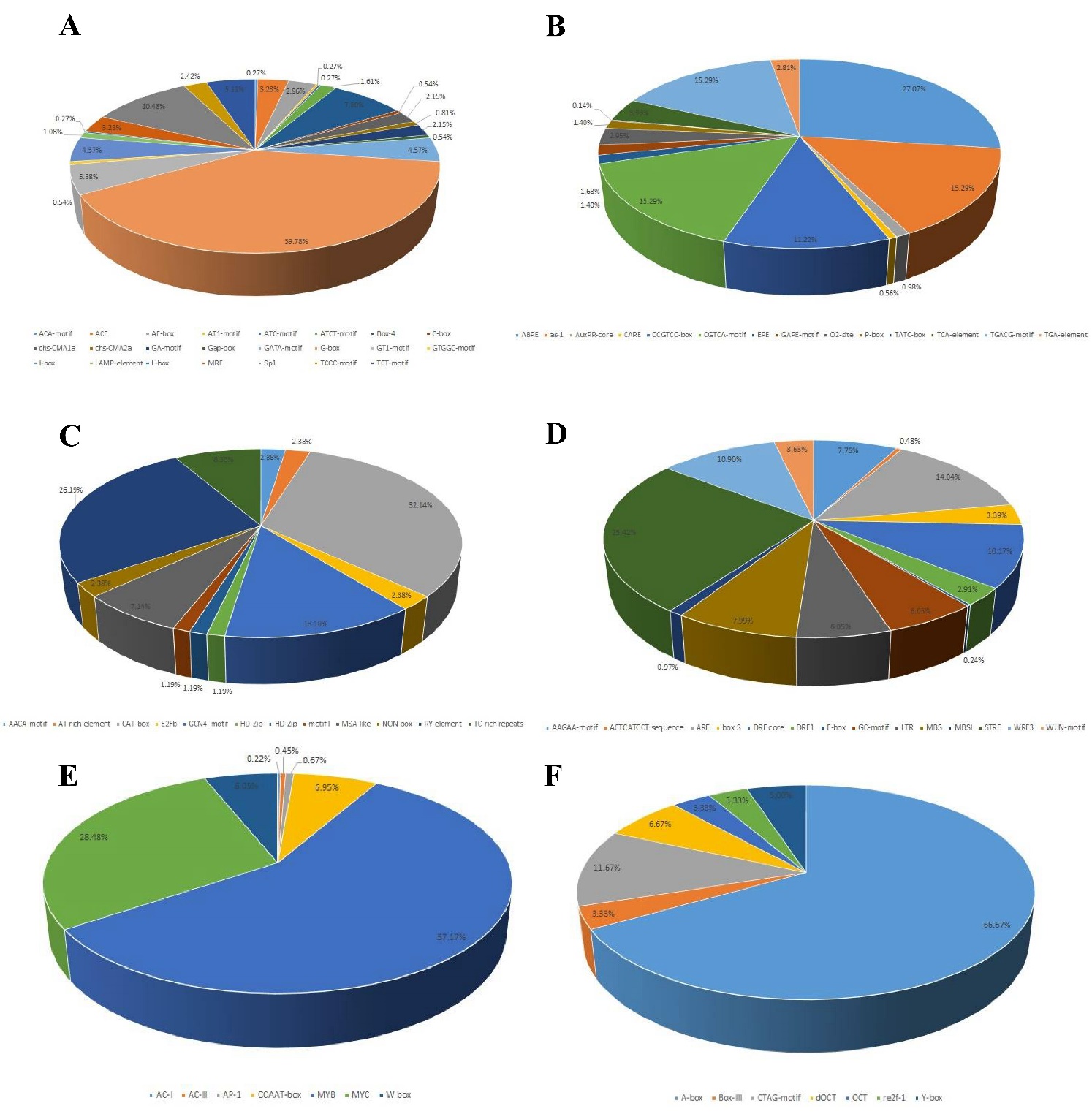
**

**Figure S4** Statistics of cis-elements found in Qingke *TLP* genes. **(A)** light responsive. **(B)** hormone responsive. **(C)** growth and development. **(D)** Stress responsive. **(E)** Transcription factor binding sites. **(F)** Unknown function.
