# Supplementary material for: Genome-wide analysis of the Thaumatin-like gene family in Qingke (Hordeum vulgare L. var. nudum) uncovers candidates involved in plant defense against biotic and abiotic stresses
Source: Front Plant Sci. 2022 Aug 17;13:912296. doi: 10.3389/fpls.2022.912296 (PMC9428612; doi:10.3389/fpls.2022.912296)
Supplement: Supplementary file 4 [file Table_3.DOCX]

**Supplementary Table S2** Primers used in the present study

| **Experiment** | **Primer name** | **Primer sequence (5′–3′)** |
| --- | --- | --- |
| *HSP* | HSP qPCR-F | AGAGCAAGATGGAGGAGGTCG |
|  | HSP qPCR-R | AGCAGATGAAAGCAATAAGCA |
| *HOVUSG6558000* qPCR | HOVUSG6558000 qPCR-F | CTTCTTCGACCTGTCCGTCAT |
|  | HOVUSG6558000 qPCR-R | GAGGACGATCTGGTAGTTTGTTC |
| *HOVUSG3036300* qPCR | HOVUSG3036300 qPCR-F | GGTGCCATAGTTGCCATAGTT |
|  | HOVUSG3036300 qPCR-R | ATTGCTCTGTTGCTGCTTCTAA |
| *HOVUSG0170200* qPCR | HOVUSG0170200 qPCR-F | CTTGGCAGAGGTTCAGACAAT |
|  | HOVUSG0170200 qPCR-R | TTCTAACGGAGGAGCAGTGT |
| *HOVUSG3065300* qPCR | HOVUSG3065300 qPCR-F | TCTTCCTCCTCCTTGCTGTT |
|  | HOVUSG3065300 qPCR-R | ATATTGTGGAGCCGCAGTTG |
| *HOVUSG5063600* qPCR | HOVUSG5063600 qPCR-F | CGCTATGGATTCCTACGACATC |
|  | HOVUSG5063600 qPCR-R | GGGCAGAAGACGACTTGAAA |
