# Supplementary material for: Genome-wide analysis of the Thaumatin-like gene family in Qingke (Hordeum vulgare L. var. nudum) uncovers candidates involved in plant defense against biotic and abiotic stresses
Source: Front Plant Sci. 2022 Aug 17;13:912296. doi: 10.3389/fpls.2022.912296 (PMC9428612; doi:10.3389/fpls.2022.912296)
Supplement: Supplementary file 5 [file Table_4.DOCX]

**Supplementary Table S3** Domain analysis of TLPs in Qingke.

| **Protein** | **Domain 1** | **Start** | **End** | **Length** | **E-value** | **Domain 2** | **Start** | **End** | **Length** | **E-value** |
| --- | --- | --- | --- | --- | --- | --- | --- | --- | --- | --- |
| HOVUSG4889300 | Thaumatin | 47 | 254 | 208 | 7E-88 | N/A | N/A | N/A | N/A | N/A |
| HOVUSG1548800 | Thaumatin | 40 | 261 | 222 | 1.1E-86 | N/A | N/A | N/A | N/A | N/A |
| HOVUSG2056400 | Thaumatin | 39 | 252 | 214 | 1.9E-86 | N/A | N/A | N/A | N/A | N/A |
| HOVUSG2715500 | Thaumatin | 31 | 264 | 234 | 3.8E-86 | N/A | N/A | N/A | N/A | N/A |
| HOVUSG2403700 | Thaumatin | 6 | 226 | 221 | 8.6E-86 | N/A | N/A | N/A | N/A | N/A |
| HOVUSG5777800 | Thaumatin | 36 | 252 | 217 | 1.2E-85 | N/A | N/A | N/A | N/A | N/A |
| HOVUSG5063300 | Thaumatin | 26 | 220 | 195 | 2.5E-66 | N/A | N/A | N/A | N/A | N/A |
| HOVUSG3013000 | Thaumatin | 30 | 245 | 216 | 1.4E-84 | N/A | N/A | N/A | N/A | N/A |
| HOVUSG2714800 | Thaumatin | 106 | 320 | 215 | 8.7E-84 | N/A | N/A | N/A | N/A | N/A |
| HOVUSG3359300 | Thaumatin | 36 | 257 | 222 | 2.3E-82 | N/A | N/A | N/A | N/A | N/A |
| HOVUSG6064000 | Thaumatin | 34 | 248 | 215 | 1.6E-80 | N/A | N/A | N/A | N/A | N/A |
| HOVUSG2056300 | Thaumatin | 34 | 252 | 219 | 1.7E-80 | N/A | N/A | N/A | N/A | N/A |
| HOVUSG5482900 | Thaumatin | 13 | 219 | 207 | 2.2E-80 | N/A | N/A | N/A | N/A | N/A |
| HOVUSG1461300 | Thaumatin | 1 | 213 | 213 | 6.8E-80 | N/A | N/A | N/A | N/A | N/A |
| HOVUSG6188700 | Thaumatin | 42 | 259 | 218 | 5.4E-79 | N/A | N/A | N/A | N/A | N/A |
| HOVUSG1336900 | Thaumatin | 57 | 268 | 212 | 5.9E-79 | N/A | N/A | N/A | N/A | N/A |
| HOVUSG3726900 | Thaumatin | 55 | 277 | 223 | 1.1E-78 | N/A | N/A | N/A | N/A | N/A |
| HOVUSG6245400 | Thaumatin | 35 | 250 | 216 | 9.7E-76 | N/A | N/A | N/A | N/A | N/A |
| HOVUSG6558000 | Thaumatin | 31 | 227 | 197 | 8E-68 | N/A | N/A | N/A | N/A | N/A |
| HOVUSG5063500 | Thaumatin | 31 | 226 | 196 | 2.6E-67 | N/A | N/A | N/A | N/A | N/A |
| HOVUSG5062800 | Thaumatin | 266 | 463 | 198 | 5.4E-66 | N/A | N/A | N/A | N/A | N/A |
| HOVUSG3065300 | Thaumatin | 27 | 173 | 147 | 1.65E-63 | N/A | N/A | N/A | N/A | N/A |
| HOVUSG5062900 | Thaumatin | 29 | 172 | 144 | 1.1E-86 | N/A | N/A | N/A | N/A | N/A |
| HOVUSG5063600 | Thaumatin | 27 | 151 | 125 | 4.14E-73 | N/A | N/A | N/A | N/A | N/A |
| HOVUSG6418000 | Thaumatin | 33 | 174 | 142 | 8.6E-86 | N/A | N/A | N/A | N/A | N/A |
| HOVUSG5872500 | Thaumatin | 28 | 168 | 141 | 1.2E-85 | N/A | N/A | N/A | N/A | N/A |
| HOVUSG4917800 | Thaumatin | 37 | 185 | 149 | 2.5E-66 | N/A | N/A | N/A | N/A | N/A |
| HOVUSG2829200 | Thaumatin | 31 | 175 | 145 | 1.4E-84 | N/A | N/A | N/A | N/A | N/A |
| HOVUSG5063200 | Thaumatin | 25 | 153 | 129 | 1.07E-51 | N/A | N/A | N/A | N/A | N/A |
| HOVUSG3036800 | Thaumatin | 1 | 159 | 159 | 1.9E-86 | N/A | N/A | N/A | N/A | N/A |
| HOVUSG3036500 | Thaumatin | 31 | 232 | 202 | 1.5E-63 | Protein kinase domain | 331 | 595 | 265 | 8.5e-50 |
| HOVUSG0169900 | Thaumatin | 25 | 227 | 203 | 3.8E-62 | Protein kinase domain | 323 | 527 | 205 | 1.3e-07 |
| HOVUSG3036300 | Thaumatin | 36 | 238 | 203 | 2.8E-56 | Protein kinase domain | 329 | 598 | 270 | 1.0e-50 |
| HOVUSG0170500 | Thaumatin | 1 | 188 | 188 | 8.2E-47 | Protein kinase domain | 344 | 482 | 139 | 7.1e-20 |
| HOVUSG0170200 | Thaumatin | 38 | 232 | 195 | 3.6E-43 | Protein kinase domain | 310 | 526 | 217 | 1.1e-46 |
| HOVUSG6545900 | Thaumatin | 353 | 567 | 215 | 2.8E-79 | Cyclin N-terminal domain | 26 | 126 | 127 | 4.8e-20 |
